# Audiovisual Media Communications in Adult Education: The case of Cyprus and Greece of Adults as Adult Learners

**DOI:** 10.3390/ejihpe10040069

**Published:** 2020-10-14

**Authors:** Constantinos Nicolaou, George Kalliris

**Affiliations:** Laboratory of Electronic Media, School of Journalism and Mass Communications, Faculty of Economic and Political Sciences, Aristotle University of Thessaloniki, 546 36 Thessaloniki, Greece; gkal@jour.auth.gr

**Keywords:** adult education, adult educator, adult learner, audiovisual media technologies, audiovisual content, teaching methodologies, non-verbal communication, lesson plan, TV, generations

## Abstract

Nowadays, audiovisual media technologies and audiovisual content (audiovisual media communications) play an important role in our physical/psychological health, education, and lifelong learning, causing the redefinition of the teaching methodology. As presented in the literature, the use of audiovisual media communications presuppose a new way of approaching effective teaching, which requires the educators on all educational levels and disciplines to display with competence many advanced skills and abilities. The aim of this research is to provide data that will contribute to the effective teaching utilizing audiovisual media communications in adult education. This research is a secondary research from two researches, which are qualitative and based on a quantitative method of analyzing. The primary data were collected through experiment method from adults (18 years and older), in Cyprus and Greece. The results confirm the current debate of using audiovisual media technologies within the educational process in technology-enhanced learning in education, both from the literature, and from the findings and results of various researches. This research is part of a larger, ongoing research that explores the multidisciplinary field that incorporates media, audiovisual content, and education (MACE), information and communications technologies (ICTs) in adult education (in Greece and Cyprus).

## 1. Introduction

The rapid development in the fields of science and technology in recent years has brought about and continues to bring about change [1,2,3], especially in adult education [4,5,6,7]. Information and communications technologies (ICTs) are essentially contributing to the enhancement and effectiveness of the provided adult education [4,5,6,7,8,9,10], which has always been at the center of social changes [1,2,3,7,8,9]. Audiovisual media technologies are integrated in ICTs and are used as educational techniques and tools to create and disseminate digital media literacy [11], employing widespread content delivery modes, which result in acquiring improved knowledge, and in order to achieve proper and constructive communication (verbal and non-verbal) [12,13], while developing skills identified through 4C: *communication*, *collaboration*, *critical thinking*, and *creativity* [14]. Effective integration of audiovisual media technologies requires dedicated and talented adult educators [15,16] who facilitate broadening the educational process from curriculum [17,18] and textbook-centered material up to real-world Internet applications and services [4,5,6,7,8,15,16,17,18,19], combining communication techniques [12,13,19,20,21,22,23] and taking into account the adult learners’ profile [15,24,25,26].

In a conventional educational environment, educators on all educational levels and disciplines should organize a prepared lesson plan prior to the presentation of a lesson to the learners (including the adult learners) [17], which is essentially a prearranged strategy for imparting information. Nowadays, educators are increasingly presenting information with the assistance of audiovisual media technologies in education to support technology-enhanced learning [15,27,28,29,30] because they believe learners learn better [18], which is documented through literature review [15,16]. On the other hand, in adult education, although there is an important and valuable literature review [4,5,6,7,15], very little research has examined the pedagogical value of using audiovisual media technologies within the educational process in technology-enhanced learning [27,28]. The new adult generations (e.g., Generation Z/GenZ from 1995 [31,32]) that are now at the educational stages approach information mainly through audiovisual media technologies [27,28,33,34] and learn in this way [15,27,28], but what about the older generations (e.g., Silent Generation from 1925 to 1945 [35,36], Baby Boomer Generation from 1946 to 1964 [37,38], Generation X/GenX from 1965 to 1979 [39,40], and Millennials or Generation Y/GenY from 1980 to 1994 [41,42,43]), and what should adult educators do? Audiovisual media technologies can be implemented by educators on all educational levels and disciplines [15], either as tools they use themselves to convey knowledge through stimulating the senses as a vivid teaching environment is created, or as tools for the learners and the adult learners to use which correlate concepts with skills to reach more effective outcomes through creativity [6,7,8,16].

This research is part of a larger, ongoing research that explores the multidisciplinary field that incorporates media, audiovisual content, and education (MACE), ICTs in adult education (in Greece and Cyprus), which began in 2016, while part of its primary data was used as secondary data with other primary data of other researches for a secondary analysis [12,13]. It consists of two researches, with a sample of adults as adult learners (18 years and older) from Cyprus and Greece (during the period 2019 to 2020). The primary data of this research were collected through the traditional experiment method (qualitative method) after or/and before conducting an interactive educational seminar based on a lesson plan using audiovisual media technologies and audiovisual content (audiovisual media communications from here on) through non-verbal communication, which were coded based on the new methodological approaches [44,45,46,47] as well as from and through Internet applications and services.

The aim of the research is to provide data that will contribute to the quality of adult education, and more specifically, the use of audiovisual media communications through teaching as educational techniques and tools to provide technology-enhanced learning, because nowadays the field of education technology (including mass media) is (still) plagued [48,49]. The purpose of the research, which was set from the beginning, was twofold. The primary objective were to investigate (a) the suitability of specific audiovisual media communications for use in any educational process (e.g., through a lesson plan); (b) the attitudes of adults as adult learners towards the specific audiovisual media communications that were used in interactive teaching (seminar)—testing of (new) theory of audiovisual media in education [15], as well as (c) whether their use of audiovisual media communications respectively helps or alters the physical/psychological condition (level of fatigue or/and tiredness) of the participants at the end of the lesson (seminar), both as a device (audiovisual media technology) [50,51,52] and as a content (audiovisual content) [53,54], as reported in the literature in relation to mass media [54,55,56,57]; and secondly, to present through research (as a case study) a lesson plan using audiovisual media communications through non-verbal communication as an exemplar for use.

In summary, these two researches that form the specific research, as well as ongoing research that explores the multidisciplinary field that incorporates MACE where included as part, is/are considered original because, (a) this methodology (as methodological approach which is part of the new research methods [44,47]) is applied for the first time, while (b) is implemented in two countries (Cyprus and Greece) at the same time. In addition, it contributes to the current debate of using audiovisual media technologies within the educational process in technology-enhanced learning [15,27,28,29,30], and especially in the field of audiovisual media in relation to adult education [4,5,6,7]. In conclusion, this article will address issues of audiovisual media, teaching methodology, and non-verbal communication, aiming at quality and efficient teaching in adult education.

## 2. Background and Literature Review

The education world today is changing enormously due the fact that everything is extremely visualized [12,13,15,16,27]. From the rapid development in the fields of science and technology in recent years to globalization [1,2,3], education today faces numerous challenges that have a significant impact on learning and teaching itself [15]. Moreover, the challenge has forced educators to think differently about teaching, resulting in the creation of new and modern teaching trends [16,58,59], such as differentiated teaching and interdisciplinary teaching which can apply technology-enhanced learning [15].

The benefits of using and applying the audiovisual media technologies (including mass media, e.g., radio [60] and television [61]) within the classroom [62] are already known, and especially in adult education where they have been known for five decades, using the (traditional) mass media [63,64,65,66]. For some unexplained reason, academia and researchers have stopped exploring the use of mass media in recent decades, as well as the audiovisual media technologies as new technologies in adult education (based on search results from the website database/search engine ERIC as specialized tool, using the terms “adult education” and “audiovisual media”, 21 results were found, of which only the 13 relevant to the topic after the systematic search of literature review, with chronologies from 1967 to 1979; at the time of writing this article). This may be due to (a) the rapid development of technology and the emergence (or re-emergence) of new teaching methods [15,16], or/and (b) because the then adult learners or/and adult educators were not ready for this technological innovation. If we consider the genealogical characteristics based on the genealogical cohorts of the then adult educators and learners (that is, they were members of the Baby Boomer Generation and especially of the Silent Generation, who are unfamiliar with the use of technology as *digital immigrants* [67,68]), indeed, the adults then were not ready to manage the use of new technologies in adult education. New technologies five decades ago were considered the (traditional) radio (1920) and television (TV from here on) (1957) [69], while today, interactive websites and weblogs/blogs, social media networks and platforms (e.g., LinkedIn, Facebook, Twitter, etc.), audiovisual platforms (e.g., YouTube, Vimeo, Netflix, etc.), Internet applications and services (e.g., Internet relay chat or messaging apps/social messaging/social chat, such as Skype, Viber, Facebook Messenger, WhatsApp, etc.), new media (e.g., Internet radio/TV or web-radio/TV), etc. [1,2,3,15]; so-called ‘ICTs’ are an umbrella term that includes basically any communication device, application, or service (as audiovisual media technologies) [15].

Nowadays, audiovisual media technologies are used as educational techniques and tools in the educational process (e.g., through the implementation of a lesson plan) [15], because they are an important factor in achieving enhanced learning, while, at the same time, playing a critical role in the success of teaching [16] as well as an important role (mainly non-verbal [12,13,22,23]) in our psychological health through the consumption of audiovisual content [54]. The literature states that the use of audiovisual media technologies presupposes new ways of approaching effective teaching [15,16,70] and also requires educators at all educational levels and disciplines to display many advanced skills and abilities with competence [15]. To be able to achieve this, educators should use them based on critical analysis and discussion of the transmitted messages as well as the self-action of learners [15]. Every learner learns differently [15,16], so does the adult learner in adult education.

Adult learners learn under certain conditions and circumstances [26,71], such as (a) when they (i) understand, realize, and accept the aims of the training program/lesson/course/seminar, (ii) act and get involved, and (iii) train in a climate conducive to participation; as well as (b) when the education has a direct relation to everyday life [4,5,72]. Thus, the adult educators should (a) teach, but also help adult learners how to learn (like students in school [73], but differently from the way they have traditionally been treated in schooling [71]); (b) provide them with the ability to continue learning; and (c) provide motivation through adult learning [4,5,26,74]. 

In the literature, it states that adult education is mainly about adults with inherent (e.g., performance, gender, religion, and age) and specific characteristics, such as people in general or/and minority populations, vulnerable social groups, and special audiences (e.g., people with muscular disabilities or kinetic problems, impaired vision) who cannot read or have a different native language [24,25,71,72], aiming to fight against cultural inequalities, exploring adult learning opportunities, and raising the general level of culture [4,5]. Moreover, that it is a vital element of the uninterrupted learning pathway because it concerns the whole range of formal, non-formal, and informal learning process as defined today [4,5], so adult educators should always innovate in the use of audiovisual media technologies [15,16,70] and differentiate the methods of didactic processes for providing quality in education to meet the criteria of each level and discipline [15,16,26,70,72,74], giving equal opportunities [24,25,71].

## 3. Materials and Methods 

The present research is considered a secondary research method, which presents primary data from two researches (something we will discuss below for each research separately), with a sample of adults as adult learners (18 years and older) from Cyprus and Greece (during the period 2019 to 2020), through the traditional experiment method (qualitative method) as classic experiment [75] (pp. 76–78) or/and quasi-experiment [76] (pp. 139–141), respectively. Although using a qualitative method (as methodology), nevertheless the two researches follow a quantitative approach to both the collection and coding and analysis of primary data through technology (including the audiovisual media technologies), and specifically from and through the Internet [47,77]; as a new methodological approach [44,45,46,47].

In the literature, the conduct of qualitative method and collecting and analyzing data through a quantitative method, is considered acceptable and is part of the field of modern mixed-method approaches [78,79], which is a common phenomenon in relevant research in the last decade [1,2,12,13,27]. Also, the specific type of methodology (i.e., the marriage as mixed-methods or exchange of roles of methods in a methodology [80,81] through technology) which was used in both researches, nowadays, it is widespread and is part of the new research methods [44,47]. This is due to the rapid development of technology and the expansion of digital technology that has led to the redistribution, re-evaluation, and reintegration of traditional research methods [44,45,46,47].

At this point, we should mention that the first research of which served as a pilot (pilot survey from here on) of the second research (main research from here on), to support and validate the main research, as defined in the literature on research methodology [76,80,82]. Also, the data (or part of the data) from the two researches were used as primary or secondary data for primary or secondary analysis respectively in relevant researches [12,13], while in the experimental method the quasi-experiment [76] (pp. 139–141) was applied.

### 3.1. Planning the Research Method: Experiment

The method used in both researches is the traditional experiment method as classic experiment [75] (pp. 76–78). The experiment in all cases was in the form of interactive educational seminar (seminar from here on) based on a lesson plan (from and through audiovisual media communications) prepared by a corresponding seminar/workshop (“Life Skills: The Importance of Non-Verbal Communication”) held in Thessaloniki in the context of the Panhellenic Conference with International Participation on “Re-Reflections on Childhood” in 2014 [23], with (a) the same theme (non-verbal communication), same teaching methodology (differentiated teaching: based mainly on the theory of constructive learning [83] and learners can learn anything at any age [84,85]); (b) the same equipment (as educational tools: computer, overhead projector/projected visuals materials via presentation software, video projection/video, speakers/sound and audio media, as well as board/marker); and (c) the same duration (90 min); applying now the theory of audiovisual media in education [15] and the sequential stages of group development [86]. All experiments were performed in different places (venues), at different times and in different periods, but all from the same adult educator/researcher. After each completion, the seminar would provide participants the necessary, not only theoretical, but mainly practical knowledge in relation to non-verbal communication at all levels, in order to be able to effectively use the techniques that compose its rich range of communication [23], as would the corresponding seminar/workshop on which it was based provide. At this point, we should mention that the original lesson plan (a) was applied as a methodological approach the edification and differentiation of teaching practices in mixed class [87], within the combination with the theoretical approaches of adult education [72] and the teaching methodology employing means of communication (audiovisual media technologies) [88], while (b) the design (of the lesson plan) was done based on curriculum development at the micro level [89] with non-verbal behavior [90] and applying theories of the motivation and personality [91] as well as the psychology of interpersonal behavior [92].

#### Lesson Plan

Improving communication skills improves the chances for our success and to respond to our communication in the way that we want. Effective communication skills through non-verbal communication allow us to develop influential techniques in real and virtual (digital) world [12,13]. 

During the seminar, learners will be (a) guided through the communication to learn more about communication skills (mostly non-verbal) [22,23] using audiovisual media communications [12,13], and also (b) practiced in a safe environment from and through the media with storytelling [16,93,94], and in particular through TV programs [54,95]. Storytelling helps learners emotionally, and allows them to construct meaning on a personal level and literacy competency [93,96,97]. At the end of the seminar, learners (in this case the adult learners) should have (a) a better understanding of the role and how to communicate effectively through non-verbal communication, and (b) exposure to, and experience of, new communication skills, presentation skills, feedback and listening skills.

The educational activities carried out applying the theory of audiovisual media in education [15] are:

1. Exercise of representation: Through a video after special processing with digital filters (sound/audio and video) through software as well as internet applications and services (mainly free, open source, cross-platform software) such as Audacity (https://www.audacityteam.org/) and Freemake Video Convert (https://www.freemake.com/), that included (a) welcome video (about the organization) with simple and panoramic shots as well as with music/songs, sound effects (sfx), as music investment, and (b) theatrical performance as role-play (is a common method in communication skills training [98]) by the adult educator (who will do the seminar) through digital storytelling and utilization of video with photos, music/song, sound and audio media, using digital filters which evoke memories or even nostalgia, feelings, affects, and emotions [54,99,100]. More specifically, the sound editing and mixing was done through WaveLab 7 (WaveLab from here on) and Audacity 2.1.3 (Audacity from here on), and the editing and production through Magisto by Vimeo 6.2.4.20511 (mobile app) (Magisto from here on), Freemake Video Convert 4.1.10 (FreeMake from here on), Movie Maker 10 (Movie Maker from here on), and YouTube Studio (Appendix A).
2. Exercise of memory activation (bringing back memories of nostalgia [101] or/and creating *willful nostalgia* [102,103,104]): Using audiovisual content through excerpts of animated movie *Sailor Moon* of Toei Animation (animation series from 1992 to 1995) with (a) the authentic and classic Greek dubbing by ‘SPK Video Film Television’ on behalf of the private Greek channel AΝΤ1 (in 1995 to 1998) (which has also been shown in Cyprus from private Greek-Cypriot channels ANT1 and VOX TV), (b) the Greek dubbing of the private Greek channel STAR (in 2001 to 2004), and (c) the Greek amateur/nonprofessional dubbing by the internet team Wings of Destiny (WoD) (https://wingsofdestiny.forumotion.net/) (since 2008); with synchronization, production, and editing by the internet team WoD as well as (a) the original Japanese dubbing by Toei Animation and (b) the American/English dubbing by DiC Entertainment (in 1995); which was also processed through software as well as Internet applications and services (sound editing and mixing via WaveLab and Audacity as well as edit and production via Magisto, FreeMake, Movie Maker and YouTube Studio).
3. Brainstorming (with “communication” and “non-verbal communication”): Using visual media such as projected visuals materials via presentation software (e.g., Microsoft PowerPoint) [105,106,107].
4. Suggestion as storytelling about the non-verbal communication through edited video (via Magisto, FreeMake, Movie Maker, and YouTube Studio) with specific well-known and famous scenes from various foreign (a) TV series, such as *Sex and the City* from HBO (1998–2004), *The Walking Dead* from ACM/FOX/Netflix (2010–), *Coven* (2013–2014), and *Apocalypse* (2018) of American Horror Story (2011–), and *9-1-1* (2018–) from FX/FOX; (b) TV productions, such as *RuPaul’s Drag Race* (2009–) from LOGOtv/VH1/Netflix, *America’s Next Top Model/ANTM* (2003–2018) from UPN/The CW/VH1, *Eurovision Song Contest 2019* by EBU (2019), and *Eye Contact* by Ten Twenty Films (2012); and (c) movies, such as *300* (2006), *Mean Girls 1* (2004), *Sex and the City 1* and *2* (2008 and 2010), *A Thousand Words* (2012), and *Clueless* (1995); as well as simple and panoramic shots from the erotic city of Thessaloniki (Greece); enriched with music/songs, sound effects (sfx), and Greek voice over/human speech through music production and editing (via WaveLab and Audacity); production-based of *Non-Verbal Communication—The Documentary* by R.O.D. Films (2010) (where excerpts were also used for the final video) (Appendix B).
5. Guided didactic discussion and learning discussions with experiential education: e.g., how non-verbal communication is imprinted based on the knowledge through their own previous knowledge and experiences [72,103], using audiovisual content through (a) excerpts from well-known Greek TV series (which have been shown or continue to be shown in Greece and Cyprus), such as (i) the sequel of the series *S’ Agapo M’ Agapas/I Love You, You Love Me* (*Σ’αγαπώ Μ’αγαπάς* of Greek language) (2000–2002) of the private Greek station MEGA (which have been also shown in Cyprus on the former private Greek-Cypriot channel MEGA—now OMEGA from 2018) from the pay-TV platform of Greek telecommunication provider COSMOTE (2019–) (the new episodes are also available through audiovisual platform YouTube), (ii) the *Sto Para 5/In the Nick of Time* (*Στο Παρά 5* of Greek language) (2005–2007), the *Ichni/Wake* (*Ίχνη* of Greek language) (2007–2008), and the *Dolce Vita* (*Ντόλτσε Βίτα* of Greek language) (1995–1997) from private Greek channel MEGA (which have been also shown in Cyprus on the former private Greek-Cypriot channel MEGA), as well as (iii) the *Konstantinou and Elenis/Constantine’s and Helen’s* (*Κωνσταντίνου και Ελένης* of Greek language) (1998–2000) from private Greek channel ANT1 (which have been also shown in Cyprus from private Greek-Cypriot channel ANT1); which was also processed through software as well as Internet applications and services (sound editing and mixing via WaveLab and Audacity as well as edit and production via Magisto, FreeMake, Movie Maker, and YouTube Studio) and (b) a video with scenes from the Greek production of *Greece’s Next Top Model 2/GNTM 2* (2019) from private Greek channel STAR (which have been shown in Cyprus from private Greek-Cypriot channel OMEGA); after special processing through video editing and production (via Movie Make, Freemake, and YouTube Studio) as well as enriched with music/songs, sound effects (sfx), and Greek voice over/human speech through music production and editing (via WaveLab and Audacity) which evoke memories or even nostalgia, feelings, affects, and emotions [54,99,100] (Appendix C).
6. Awakening (using the online game-based learning platform “Kahoot!”—free online learning platform/interactive website—creating a test for non-verbal communication, in order to add vitality, learners engagement, and meta-cognitive supports because the game promotes motivation and in particular this technology enhances learning [108,109]) and plenary debate.
7. Meta-cognitive knowledge and evaluation meta-cognitive skill (using the first video—second part—of the theatrical performance by the adult educator).

### 3.2. First Research: Pilot Survey

#### 3.2.1. Sample and Method

The pilot survey of the main research (conducting pilot experiments) started in March 2019 and was completed in January 2020 in three phases (experiments), involving 40 adults from Cyprus and Greece as trainee volunteers (adult learners): (a) 14 adults (teachers from secondary education) from various cities and regions of Cyprus (e.g., Nicosia/Lefkosia, Limassol/Lemesos, Larnaca/Larnaka, Paphos/Pafos, Famagusta/Ammochostos, etc.) who attended the seminar/workshop (“The (non-verbal) communication (to the solution) of conflicts”) in the context of the 18th Pancyprian Scientific Conference of the Educational Group of Cyprus (Εκπαιδευτικός Όμιλος Κύπρου/ΕOΚ of Greek language) on “RE-view of the Public School of Cyprus in a World of Constant Changes and Challenges” in Limassol/Lemesos (March 2019) [22] (first pilot phase); (b) nine adults from Thessaloniki (Greece) (five PhD candidates and four postgraduate students of the School of Journalism and Mass Communications, Faculty of Economic and Political Sciences, Aristotle University of Thessaloniki) (October 2019) (second pilot phase); and (c) 17 adult educators from Athens (Greece) who attended the pilot program of Adult Educator Training (200 h) with mixed learning (164 h of distance education through mass open online course (MOOC) and 36 h of lifelong education) from the “European Agenda for Adult Education and Training” in Greece undertaken by the General Secretariat for Lifelong Learning and Youth (Greek Ministry of Education and Religious Affairs), in order to pass the examinations for certification as accredited ‘Trainers for Adults’ in the “National Organization for the Certification of Qualifications (in the official abbreviation for English language) and the National Centre for Vocational Guidance (EKEP in the official abbreviation for English language)” (EOPPEP in the official abbreviation for English language) (January 2020) (third phase). The sample used in the pilot survey through the traditional experiment method based on literature is considered acceptable [80,81] and conceptually valid [76] (pp. 143–145). Finally, the primary purpose of the pilot survey was the cultural adaptation of the main research and the feedback through a form as evaluation (to be discussed below).

#### 3.2.2. Design and Creation of the Data Collection Form

The data collection was carried out through a specially designed written questionnaire in the form of an evaluation (data collection form from here on), in quantitative (using the Likert scale [110,111]) and qualitative format, which was passed to the participants after the completion of the seminar (experiment).

The “data collection form” was divided into two parts: (a) the first part, contained six questions with a five-point Likert scale in relation to the degree of satisfaction of quality (1 = ‘Very Poor’ to 5 = ‘Excellent’) for the “expectations”, the “organization”, the “interesting suggestions”, the “development issues” (if they were interesting), the “knowledge” acquired (theoretical background investigation), and the “equipment” (as educational tools) used; and (b) the second part, one open-ended question in relation to the views, provide feedback, retrieve the problems, and evaluate possible solutions of the participants about the seminar through audiovisual media communications (as comments or/and suggestions). Finally, it should be mentioned here that the “data collection form” served as *testing* [82] (p. 204) for one of the two forms used in the main research (to be discussed below).

### 3.3. Second Research: Main Research

#### 3.3.1. Sample and Method

The individuals who participated in this research (main research) as adult learners were selected through a special list of volunteer adult educators and adult learners from Greece and Cyprus, applying the rules of Internet sampling [47] (pp. 59–63). These individuals had declared their participation through an expression of interest (ΕοΙ) [77] in order to participate in a pilot research (with pilot surveys) or researches (such as this research) in February and August 2019, in an ongoing research that explores on the multidisciplinary field that incorporates MACE, which began in 2016. The list was created through the specialized online platform Survs.com as an online/electronic EoI, which was (a) posted as an announcement in relevant online groups of adult educators and adult education of a well-known social media (specifically in Facebook) (February and August 2019); and (b) sent through electronic mail (email) to all the educators of the *AGO* (*AΓO*—*Aθλητισμός για όλους* of Greek language)/*Sports for all* program of the Cyprus Sports Organization/CSO (Κυπριακός Oργανισμός Aθλητισμού/ΚOA of Greek language) in Cyprus where their emails were available on the official website of the program (https://ago.org.cy/) (August 2019), with all relevant information about the research (e.g., methods used and how it will be conducted, especially if training or education is included because we are dealing with adults [26,72]); following (i) the suggested techniques [3]; (ii) the Internet research ethics [47] (pp. 41–57) and privacy issues [77]; as well as (iii) the relevant European provisions on the use of personal data (General Data Protection Regulation—GDPR). The main research began in February 2020 and was completed at the end of June 2020, using the traditional experiment method, through four experiments (as seminars).

The sample from the final list at the end of September 2019 (when the online/electronic ΕοΙ closed) was 1363 participants, of which 1052 were adult educators and 311 were adult learners. More specifically, of the 1052 adult educators, 669 (209 were males with a percentage of 31.2% and 460 females with a percentage of 68.8%) had chosen through the EoI they were interested in participating in the main research as adult learners. The specific sample was from different regions of Greece and Cyprus and with different age groups, who worked (or work) in different structures/institutions of adult education, and in different school years. 

The adult educators who would participate in the main research had to meet certain conditions, such as (a) being active adult educators during the school year 2019–2020, and (b) being available to attend the specific day that the research would take place (February 2020 for Athens and Thessaloniki in Greece as well as June 2020 for Nicosia/Lefkosia and Limassol/Lemesos in Cyprus), after a relevant communication through phone or short message service (SMS), text message, or email through specialized online platform (e.g., web SMS/email page or web SMS/email API, an enterprise-class service to send SMS/email using software such as cyta.com.cy), applying the relevant privacy issues in Internet research (including the rules of Internet sampling through an ΕοΙ) [77]. In addition, for obvious reasons, it was decided to select samples from only the two larger cities of each country, respectively (Athens and Thessaloniki from Greece as well as Nicosia/Lefkosia and Limassol/Lemesos from Cyprus), as well as from specific structures/institutions of adult education and training (Public Vocational Training Institute undertaken by the General Secretariat for Lifelong Learning and Youth in the Greek Ministry of Education and Religious Affairs from Greece, as well as Adult Education Centers in the Cyprus Ministry of Education, Culture, Sport, and Youth from Cyprus). Based on the mentioned criteria, the final sample of the main research consists of 76 active adult educators as adult learners, 38 participants from Greece with a percentage of 50% (19 adult educators from Athens with a percentage of 25% and 19 adult educators from Thessaloniki with a percentage of 25%), and 38 participants from Cyprus with a percentage of 50% (19 adult educators from Nicosia/Lefkosia with 25% and 19 adult educators from Limassol/Lemesos with 25%). The specific sample, based on the literature is conceptually valid [76] (pp. 143–145) and considered acceptable [80,81] to be the sample (≥15) in a research that uses the experiment method [80] (p. 102), as well as based on the rules of Internet sampling [47] (pp. 59–63).

#### 3.3.2. Design and Creation of the Data Collection Form: Research Protocol

The data collection was carried out through a research protocol; (a) a questionnaire assessing general fatigue measurement scale respectively, which was completed before and after the seminar (experiment), and (b) a specially designed written questionnaire in the form of an evaluation form, in quantitative (using the Likert scale [110,111]) and qualitative format, which was passed to the participants after the completion of the seminar (experiment).

The fatigue questionnaire was the Fatigue Severity Scale or FSS [112] (FSS form from here on), which consists of nine questions (items) (Appendix D). It was developed to measure fatigue in patients with multiple sclerosis (MS) and systematic lupus erythematosus (SLE) [112], while subsequent has been widely used in various psychometric studies. The items are scored on seven-point Likert scale (1 = ‘strongly disagree’ to 7 = ‘strongly agree’). The best way of scoring is mean of all the scores with minimum score being 1 and maximum score being 7. The “FSS form”, although available in Greek (after the academic method of translation and compensation) [113,114], in this case the proposed procedures and strategies for rendering culture-specific concepts (CSCs) [115] and allusions respectively were followed. Specifically, the translation process was applied as proposed by the Scientific Advisory Committee (SAC) of Medicine [116,117] in combination with the practices proposed by a team at International Society for Pharmacoeconomics and Outcomes Research (ISPOR) [118]. 

The specially designed written questionnaire (evaluation form from here on) was divided into three parts: (a) The first part, contained 11 questions (including the six questions used in the “data collection form” for the pilot survey) with a five-point Likert scale in relation to the degree of satisfaction of quality (1 = ‘Very Poor’ to 5 = ‘Excellent’) for the “expectations”, the “organization”, the “interesting suggestions”, the “discussion time”, the “development issues” (if they were interesting), the “questions/answers” at the end of the seminar, the “knowledge” acquired (theoretical background investigation), the “time” (time conducted), the “venue”, the “period” (that took place), and the “equipment” (as educational tools from here on) used; (b) the second part, one open-ended question in relation to the views, provide feedback, retrieve the problems, and evaluate possible solutions of the participants (adult educators) about the seminar through audiovisual media communications (as comments or suggestions), and finally (c) the third part, questions about the profile and demographics of the sample.

After this process was completed (creation of questionnaires/forms) and before the final main research was carried out, another pilot survey (pilot study from here on) was conducted to ascertain the effectiveness of all the questionnaires (“evaluation from” and “FSS form”), and identify or correct any errors in order to make the necessary corrective interventions in time, as well as regarding cultural adaptation. The pilot study was also carried out as a method of checking the validity of the main research. In this case, 22 active adult educators from different cities of Cyprus (Nicosia/Lefkosia and Limassol/Lemesos) and Greece (Thessaloniki, Drama, Alexandroupoli and Heraklion—Crete) (12 educators from Cyprus and 10 educators from Greece) were selected as participants in the pilot study, using the interview method through conventional and new media [44,45,46,47]. Participants were selected through the special list for EoI in the research on the multidisciplinary field that incorporates MACE (mentioned above) at the end of February 2019, while the final sample used based on literature is considered acceptable [80,81,119]. The pilot study started in March 2019 and concluded in April 2019, and the conclusions drawn from the interviews with the participants are that: (a) there were no ambiguities in the formalities of the questions of all the questionnaires, and (b) the questionnaires did not cause fatigue or irritation as it did not take more than 5 min (on average) to complete it. 

### 3.4. Data Processing and Analysis

The data were coded based on the new methodological approaches [44,45,46,47] as well as from and through Internet applications and services. Specifically, they were inserted in IBM Statistical Package for Social Sciences (SPSS) (version 20), as well as through the specialized online platform Survs.com. Before performing the analysis, the internal reliability of quantitative query data was tested using the “Cronbach’s alpha” index through SPSS, both in the pilot survey and the main research. More specifically, the internal reliability (a) of all the quantitative questions (six questions) from data collection form of the pilot survey is α = 0.841, and (b) of all the quantitative questions (29 questions) from research protocol of the main research is α = 0.869, while for the quantitative questions of each questionnaire respectively it is (i) α = 0.815 for the quantitative questions (nine questions) of the “FSS form” completed before the conduct of the seminar, (ii) α = 0.886 for the quantitative questions (11 questions) of the “evaluation form” completed at the end of the seminar, and (iii) α = 0.788 for the quantitative questions (nine questions) of the “FSS form” which was completed again for the second time at the end of the seminar, respectively. Based on the reported values “α” the data of the questions are characterized as reliable and thus provide the assurance of the internal reliability of the data [120], both regarding the whole research (pilot survey and main research, respectively) and on each questionnaire (“data collection form”, “evaluation form”, “FSS form”), respectively. Also, a “test-retest” method through SPSS was applied on the data of the questions of the “FSS form” from research protocol of the main research, since it was completed two times by the participants, before and at the end of the seminar. Consequently, the relevant values of “intraclass correlation coefficient” (ICC) from the nine questions (Q1 = 0.716, Q2 = 0.709, Q3 = 0.801, Q4 = 0.643, Q5 = 0.513, Q6 = 0.793, Q7 = 0.663, Q8 = 0.864, and Q9 = 0.702) are considered acceptable, since they are ≥0.40 [121], and therefore reliable and creditworthy.

The data of the question from the “evaluation form” from research protocol of the main research regarding the degree of satisfaction for the “educational tools” used in the seminar was analyzed using the following methods: (a) analysis of variance in two factors (two-way ANOVA) (2Χ2) through SPSS in relation to gender and country origin of the sample, and (b) analysis of variance in three factors (tree-way ANOVA) (2Χ3) through SPSS in relation to the country, gender, and age of the sample. The presentations of the independent variables with respect to the analysis of variance are presented in diagrams through SPSS.

All results of this research are presented in the next section as a whole or individually or in single tables or in double entry tables with percentages or rounded percentages, averages (mean values), or standard deviations (SD) after analysis through SPSS and Survs.com. Also, the qualitative data collected from the second part of (a) the “data collection form” from pilot survey and (b) the “evaluation form” from research protocol of the main research, were grouped and adjusted as quantitative data, and presented in graph through Microsoft Office Excel 2007, for better understanding [122]. The data from the “FSS form” from research protocol of the main research were analyzed and presented based on the proposed scale score in the literature [112,113,114]. Finally, the analysis commenced with descriptive statistics.

## 4. Results

### 4.1. First Research: Pilot Survey

The grouped total responses of the sample in terms of the degree of satisfaction from the “data collection form” ranged mainly in the choices of the five-point Likert scale from 4 to 5 (“Good” to “Excellent”). The two largest percentages in option 5 (“Excellent”) were collected by (a) the question for the “educational tools” used in the seminar (28 adult learners with a percentage of 70%) and (b) for the “expectations” (26 adult learners with a percentage of 65%) (Table 1).

Regarding the averages, the question which gained the highest percentage was regarding again the “educational tools” used in the seminar (mean value: 4.50, SD: 0.934), while the lowest percentage was regarding the “organization” (mean value: 4.15, SD: 0.736) (Table 1). Also, the second highest percentage based on the average was the question about the “expectations” (mean value: 4.45, SD: 0.904) (Table 1). 

From the reduction of the rounded percentages based on the country of origin of the sample, we see that the answers of adult learners from Cyprus are more positive on a five-point Likert scale in relation to the answers of adult learners from Greece (Table 2). Specifically, we see this attitude more strongly mainly on option 5 (“Excellent”) of the five-point Likert scale, as well as in the averages of each variable respectively (Table 2).

**Figure 1:**
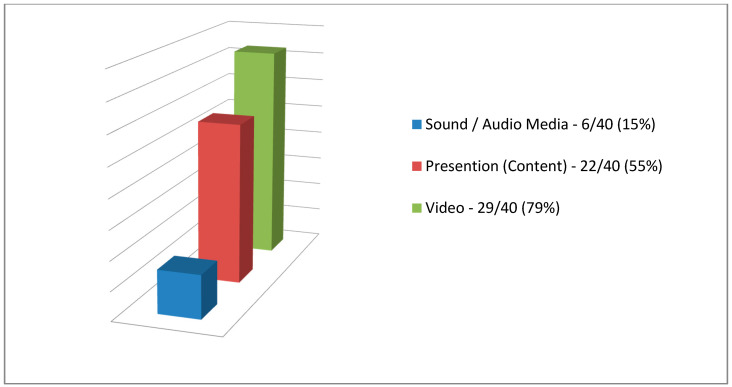
Grouped and adjusted qualitative data as quantitative data from the second part of the “data collection form” of pilot survey.

In the open-ended question, where the participants recorded their views on the seminar (as comments or/and suggestions), they mentioned (a) the use of audio spots/audio files (as sound and audio media) with a percentage of 15% (six adult learners out of 40) (audiovisual media communications), (b) the use of video with a percentage of 73% (29 adult learners out of 40) (audiovisual media communications), and finally (c) the content of the seminar presentation with a percentage of 55% (22 adult learners out of 40) (audiovisual content) (Figure 1).

### 4.2. Second Research: Main Research

The sample of adult educators, who participated in the main research as adult learners, was 76 active adult educators (adult learners from here on in this subsection) from Greece (Athens and Thessaloniki) and Cyprus (Nicosia/Lefkosia and Limassol/Lemesos) during the period 2019–2020. The statistical distribution of the variable of gender was 37 males with a percentage of 48.7% (19 males in Greece with a percentage of 51.4% and 18 males in Cyprus with a percentage of 48.6%) and 39 females with a percentage of 51.3% (19 females in Greece with a percentage of 48.7% and 20 females in Cyprus with a percentage of 51.3%). More specifically, (a) the 19 males from Greece are nine from Athens with a percentage of 47.4% and 10 from Thessaloniki with a percentage of 52.6%, and (b) the 18 males from Cyprus are 11 from Nicosia/Lefkosia with a percentage of 61.1% and seven from Limassol/Lemesos with a percentage of 38.9%, while (c) the 19 females from Greece are 10 from Athens with a percentage of 52.6% and nine from Thessaloniki with a percentage of 47.4%, and (d) the 20 females from Cyprus are eight from Nicosia/Lefkosia with a percentage of 40% and 12 from Limassol/Lemesos with a percentage of 60%.

**Figure 2:**
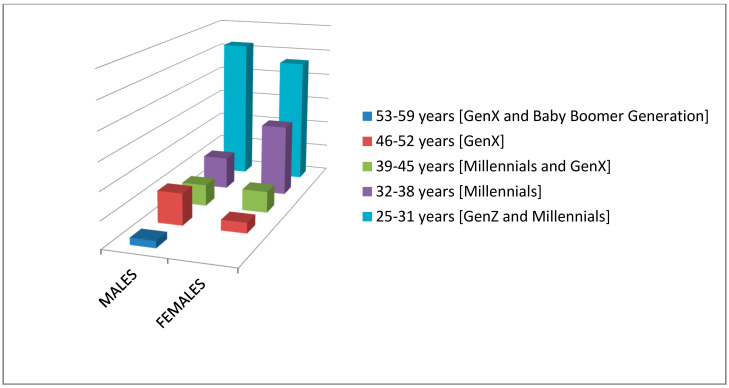
The statistical distribution of the sample of adult educators based in age groups, gender, and genealogical cohort of the main research.

Their age groups were; (a) 21 adult learners 25–31 years with a percentage of 27.6% (11 males with a percentage of 52.4% and 10 females with a percentage of 46.6%) (GenZ and Millennials), (b) 29 adult learners 32–38 years with a percentage of 38.2% (nine males with a percentage of 11.9%/31% and 20 females with a percentage of 26.3%/69%) (Millennials), (c) 12 adult learners 39–45 years with a percentage of 15.8% (six males with a percentage of 7.9%/50% and six females with a percentage of 7.9%/50%) (Millennials and GenX), (d) 12 adult learners 46–52 years with a percentage of 15.8% (nine males with a percentage of 11.85%/75% and three females with a percentage of 3.95%/25%) (GenX), and (e) two adult learners 53–59 years with a percentage of 2.6% (two males with a percentage of 2.6%/100%) (GenX and Baby Boomer Generation) (Figure 2).

The grouped total responses of the sample in terms of the degree of satisfaction from the “evaluation form” ranged mainly in the choices of the five-point Likert scale from 3 to 5 (“Fair” to “Excellent”). The two largest percentages in option 5 (“Excellent”) were collected by (a) the question for the “questions/answers” at the end of the seminar (54 adult learners with a percentage of 71%) and (b) for the “educational tools” used in the seminar (43 adult learners with a percentage of 57%) (Table 3).

Regarding the averages, the question which gained the highest percentage was regarding the “questions/answers” at the end of the seminar (mean value: 4.46, SD: 1.064), while the lowest percentage was regarding the “venue” (mean value: 3.66, SD: 0.776) (Table 3). Also, the second highest percentage based on the average was the question about the “educational tools” used in the seminar (mean value: 4.41, SD: 0.751) (Table 3). One of the most interesting results from the analysis was the average percentage regarding the “knowledge” they acquired (theoretical background investigation) on the subject (mean value: 3.97, SD: 0.894) (Table 3), something that is purely subjective for each individual [123]. In addition, we see that the degree of satisfaction in terms of “time” (mean value: 3.92, SD: 0.744), “venue” (mean value: 3.66, SD: 0.776), and “period” (mean value: 4.05, SD: 0.746) of the seminar have the lowest percentages compared to the other questions, while the choices on the five-point Likert scale ranged mainly on the choices from 2 to 4 (“Poor” to “Good”) (Table 3).

From the reduction of the rounded percentages based on the country of origin of the sample (Table 4) and gender (Table 5), we see that the answers of adult learners from Cyprus are more positive on a five-point Likert scale in relation to the answers of adult learners from Greece (Table 4), as well as females versus males, respectively (Table 5). Specifically, we see this attitude more strongly mainly on option 5 (“Excellent”) of the five-point Likert scale, as well as in the averages of each variable respectively (Table 4 and Table 5).

**Figure 3:**
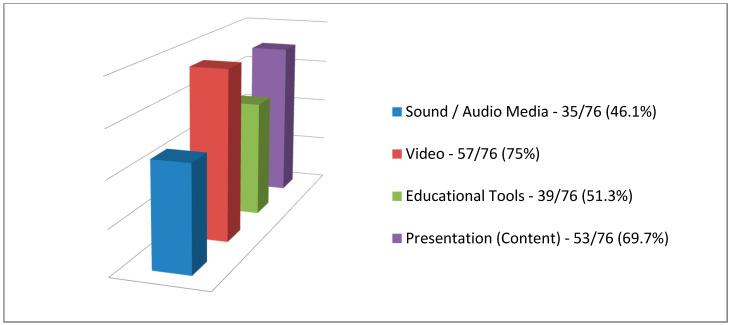
Grouped and adjusted qualitative data as quantitative data from the second part of the “evaluation form” of the main research.

In the open-ended question, where the participants recorded their views on the seminar (as comments or/and suggestions), they mentioned (a) the use of audio spots/audio files (as sound and audio media) with a percentage of 46.1% (35 adult learners out of 76) (audiovisual media communications), (b) the use of video with a percentage of 75% (57 adult learners out of 76) (audiovisual media communications), (c) the educational tools (e.g., projected visuals materials via presentation software) with a percentage of 51.3% (39 adult learners out of 76) (audiovisual media communications), and finally (d) the content of the seminar presentation with a percentage of 69.7% (53 adult learners out of 76) (audiovisual content) (Figure 3).

**Figure 4:**
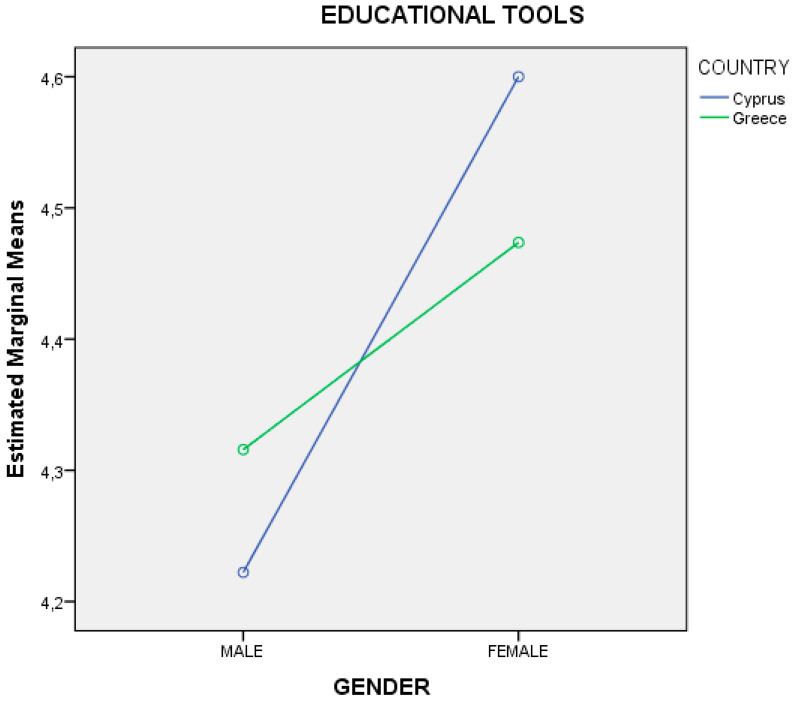
The presentation of the independent variables in two factors (two-way ANOVA) (2Χ2) in relation to gender and country origin of the sample of the main research.

The analysis of the variation on two factors (2X2) showed that the sample of Cyprus has statistically significantly higher averages than the sample of Greece in the degree of satisfaction for the “educational tools” used in the seminar, which is also shown in Table 4. The results also showed that there is a statistically significant interaction between the gender and the country of origin of the sample, something we also see in Table 5. After checking the averages, it was found that although females in Cyprus (mean value: 4.60, SD: 0.598) and Greece (mean value: 4.47, SD: 0.697) had higher averages than males in Greece (mean value: 4.32, SD: 0.885) and Cyprus (mean value: 4.22, SD: 0.808), the difference in the averages between females in Cyprus and Greece was greater (0.13) than in males between Greece and Cyprus (0.10). The interaction between the gender and the country of origin of the sample in the degree of satisfaction for the “educational tools” used in the seminar is presented in the diagram below (Figure 4). 

**Figure 5:**
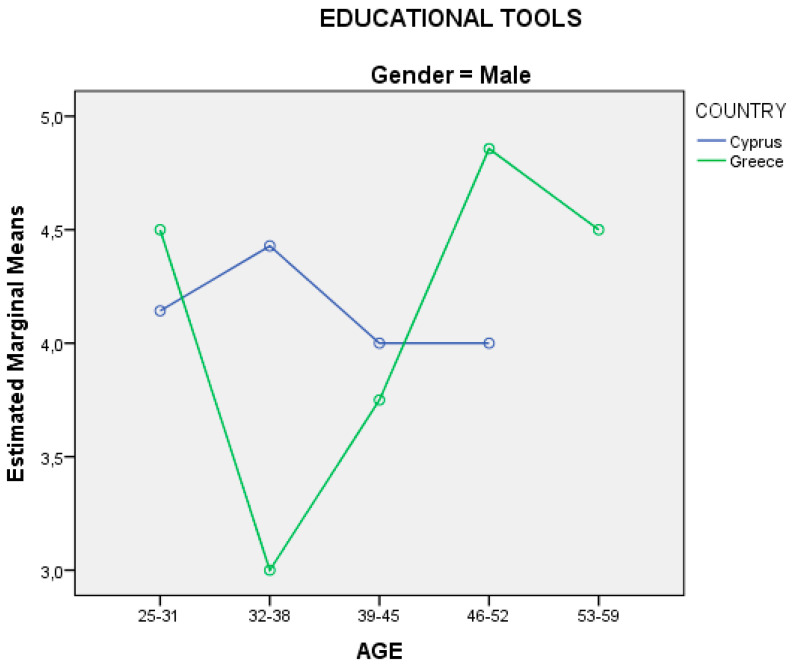
The presentation of the independent variables in three factors (tree-way ANOVA) (2Χ3) in relation to the country, gender (male), and age of the sample of the main research.

**Figure 6:**
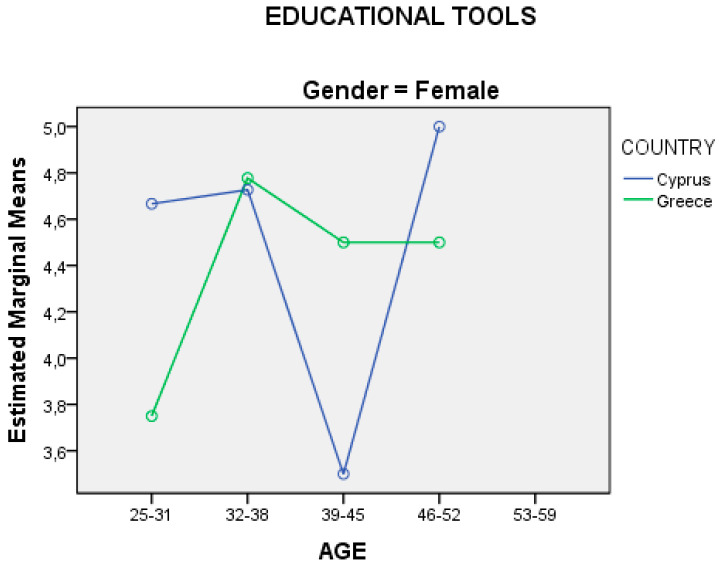
The presentation of the independent variables in three factors (tree-way ANOVA) (2Χ3) in relation to the country, gender (female), and age of the sample of the main research.

From the analysis of the variation in three factors (2X3), the results showed only one statistically significant effect, that of age. The interaction between age and the country of origin of the sample in the degree of satisfaction for the “educational tools” used in the seminar is presented in two diagrams based on gender, for male (Figure 5) and female (Figure 6).

The data collected from the questions regarding the “FSS forms” based on the relevant reliability check of “test-retest” are considered reliable. The values of “α” and “ICC” (Table 6) for the questions of the form (completed before and after the seminar) based on relevant literatures are considered reliable [121,122]. The analysis showed that the fatigue of the participants after the interactive seminar with the use of audiovisual media communications was significantly reduced (7.95%) (Table 6). In conclusion, the use of the specific audiovisual media communications can be said to reduce symptoms of fatigue or and tiredness, they improve concentration, and help people (adult learners in this case) in their psychological health.

## 5. Discussion

Nowadays, when everything is extremely visualized [12,13,15,16,27], the key for the future of adult education is ICTs [4,5,6,7,8,9,10]. Audiovisual media communications are integrated in ICTs and are used as educational techniques and tools to create and disseminate digital media literacy [11], employing widespread content delivery modes, which results in acquiring improved knowledge, and in order to achieve proper and constructive communication (verbal and non-verbal) [12,13], as well as to develop the skills identified through the 4Cs [14].

This article presents through the traditional experiment method data obtained from Cyprus and Greece, which contribute to the quality of adult education. Therefore, the sample of the research consists of adults (18 years and older), and in particular from two researches during the period 2019 to 2020: (a) 40 adults as adult learners (pilot survey) and (b) 76 active adult educators as adult learners (main research). The primary data of this research were collected (a) from March 2019 until January 2020 (pilot survey) and (b) during the first half of 2020 (February 2020 until June 2020) (main research); before and after conducting an interactive educational seminar, which were then processed and coded. 

Based on the results, we see that the aim and the twofold purpose of the research have been achieved, and the results are in accordance with the literature on the use of audiovisual media technologies in education (as well as in adult education) [4,5,6,7,8,9,10,15,16] to support technology-enhanced learning [15,27,28,29,30]. More specifically, based on the primary objective:

(a) We can safely say that the specific audiovisual media communications used through the lesson plan (i) are considered appropriate based on the answers to the open-ended questions of both in the pilot survey with adults as adult learners in Figure 1 and the main research with adult educators as adult learners in Figure 3 (researches from here on) were grouped and adjusted as quantitative data; and (ii) can be used in any educational process;
(b) The attitudes of adults as adult learners from the researches towards the specific audiovisual media communications (as educational tool) that were used in the interactive teaching (seminar), based on their answers in Table 1 and Table 3, we can safely state that the application of (new) theory of audiovisual media in education [15] is considered the most appropriate. More specifically, the specific audiovisual media technologies (computer, overhead projector/projected visuals materials via presentation software, video projection/video, and speakers/sound and audio media) and the teaching methodology (differentiated teaching which is mainly based on the theory of constructive learning [83]) are included in the theory of audiovisual media in education [15], and finally;
(c) Based on the results, the use of the specific audiovisual media communications can be said to reduce symptoms of fatigue or tiredness (physical/psychological condition), improve concentration, and help people (adult learners in this case) in their psychological health (Table 6), as stated in the literature about the benefits they offer to our psychological health which are numerous [50,51,52,53,54]. This result may be due to the audiovisual content [54] displayed through the audiovisual media technologies (something we will discuss below). If this is the case then the specific results from the main research in Table 6 contradict the findings and results of other researches which state and indicate that the content from the media are producing adverse effects on the psychological wellbeing and waning in their mental and psychological health [55,56], while reducing our physical health if more use (i.e., audiovisual consumption) [57]; this is something that would be good to investigate further.

On the other hand, based on the second objective, which “was to present through research (as a case study) a lesson plan using audiovisual communication through non-verbal communication, as an exemplar for use,” we can safely say that this lesson plan is considered reliable, based on (a) the results from this research (Section 4), (b) the relevant literature for the creation of a lesson plan through audiovisual media communications applying non-verbal communication [15,16,17,18,19,20,21], and (c) the modern approaches in teaching methodologies [124,125,126,127,128] and to teaching methodology on the use of audiovisual media technologies in adult education [4,5,6,7,8,9,10,15,16] to support technology-enhanced learning [15,27,28,29,30]; and can be used as such or part thereof by anyone.

In addition, this article refers to the generations, and in particular the last five generational cohorts (Silent Generation, Baby Boomer Generation, GenX, Millennials, and GenZ). The reason was to reinforce the conclusions of the research, and not to research the generations in adult education (but adults from 18 years and older); which in this case was impossible because the age groups of the adult educators who participated in the main research were not based on the generational cohorts such as defined in the generational theory. Based on the generational theory [39,129,130] people of the younger generations tend to perceive and use technology more actively, especially members of GenZ and Millennials who are *digital natives* in a technological digital world [67,68]. Most of the adult educators who participated in the main research as adult learners are members of the mentioned generations based on the generational cohorts (Figure 2), which is justified as *digital natives* if we consider that the individuals who participated were selected through a special list following an online/electronic EοI where it was (a) published as an announcement in relevant online groups in social media, and (b) emailed. Also, the results of the experiments showed that they kept the interest and attention of the adult educators as adult learners (Table 3), something that was a risk to keep in mind their genealogical characteristics, and especially how the younger generations consume audiovisual content [15,27,28,33,34,131,132,133]. This leads us to the conclusion that younger adult educators or/and the younger generations (GenZ and Millennials) (younger adults) adopt new technological innovations easily, since the consumption of audiovisual content in contemporary ways and platforms compared [15,27,34] to the older generations (older adults), which confirms why research for the use of media in education has ceased in the last five decades. Furthermore, we can now say with absolute confidence that the use of audiovisual media communications in adult education, but also in education in general, is necessary and required, especially from now on, something that the adult educators should always keep in mind, if they want to match the current reality [15].

Remarkably, the results also show that the use of TV programs (e.g., animated movie, TV series, TV productions, and movies) as videos (audiovisual media communications) in the educational process (in this case through the lesson plan) in technology-enhanced learning in adult education, kept the interest and attention of the participants as adult learners (Table 1 and Table 3), in both researches, and we can say that it is considered helpful for adult learners to make sense and understand the lesson (seminar), as in the relevant literature [15,16,17,27,134,135,136]. Here we should mention that the use of video in the educational process in technology-enhanced learning has a dual use, as audiovisual media technology and as audiovisual content [15,16]. The fact that the video kept the interest and attention of the participants may be due more to its use as audiovisual content and not so much as audiovisual media technology. The specific TV programs that were used as audiovisual content through the use of video, may have played a key role in the successful conduct of the interactive teaching (seminar) in relation to the age or/and genealogical cohort of the participants as adult learners, in both researches, and especially in the main research that we know the age group of the participants. Most foreign TV series, TV productions, and movies have been shown or/and are being shown (at the time of writing this article) both in Cyprus and Greece through the public or private channels of the country or/and through pay-TV platforms of telecommunications providers (e.g., CYTAVISION, COSMOTE, etc.). Also, most of them are now available through the audiovisual platform Netflix, and from 2018—when Greek subtitles became available—have started to have more recognition in both countries [137], especially in the members of GenZ and Millennials [33,34,131,132,133]. The Greek TV series used in the seminar, although they are from past decades, are still shown repeatedly on TV in both countries until today (at the time of writing this article) with a big impact in viewership as well as on the Internet through the service Web-TV or Video on Demand (VoD) platform of the channels or/and through audiovisual platform YouTube; something which is also confirmed through literature review and various researches in both countries [131,133,138,139,140]. More specifically, (a) the *Sto Para 5/In the Nick of Time* (2005–2007) and the *Dolce Vita* (1995–1997) which are still shown in Greece, as well as (b) the *Konstantinou and Elenis/Constantine’s and Helen’s* (1998–2000) which has never stopped being shown repeatedly in both countries (at the time of writing this article); resulting in familiarity to the younger adults (e.g., GenZ) who participated in this research, especially in the main research. Also, the Greek TV series *S’ Agapo M’ Agapas/I Love You, You Love Me*, which is based on the Canadian TV series *Un Gars, Une Fille/A Guy, A Girl* from Radio-Canada (1997–2003) and it was broadcast for two seasons (2000–2002), had a big impact in viewership in both countries, led the Cyprus Broadcasting Corporation (CyBC) to get the rights and make the Greek-Cypriot version of the series using the Cypriot dialect under the title *Ego Kai Esy/Me and You* for three seasons (2010–2013) on public Cypriot channel RIK1 (or CyBC1) and on the CyBC VoD platform [140]. The repercussion of this series, in whatever country it has been shown, was due to the fact that it focuses on main social issues of contemporary interest that were related to everyday life [140]. The sequel of the Greek version of the series in 2019 made the old ones remember it through nostalgia [99,101], while the younger ones learned it if they did not know it. In summary, all the above-mentioned Greek TV series are based on the triptych *love*, *friendship*, and *family* while they were adapted to the daily reality of the viewers [139,140]. All this leads us to the conclusion that the successful conduct of the seminar, may have been due to its content which was already familiar and current, something which it is confirmed through the literature that says that adult learners learn when the education has a direct relation to everyday life [4,5,72]. As a final point here, we should mention that the big impact in viewership of Greek and Greek-Cypriot TV series of the past decades is already known in recent years and a significant number of researchers have begun to study it in both countries [138,139,140,141].

In addition, from the results in Table 2 and Table 4 as well as the analysis of the variation on two factors (2X2) in Figure 4, show that the sample of Cyprus has statistically significantly higher averages than the sample of Greece in audiovisual media communications. This may be justified from the point of view of audiovisual content through relevant researches conducted in both countries: (a) in Cyprus (i) on students aged 11–13 years old in 1995 (now 36–40 years old/Millennials) with a sample of 400 students of both genders, from the whole of Cyprus [142], which safeguarded the balanced representation of the Cyprus population as to the relevant age groups; and (ii) on students aged 13–18 years old in 1997 (now 36–40 years old/Millennials), with a sample of 602 students (out of 52,900) of both genders, from the whole of Cyprus which is considered generalization to the population based on sampling followed [143], as well as (b) in Greece on students aged 9–18 years old in 2009 (now 20–29 years old/GenZ and Millennials) with a sample of 775 students of both genders, from various regions of Greece (Attica, Crete, and Ko) [144]. The results showed that the then young Greek-Cypriot students in both researches had a special preference and close relationship with TV [142,143], something which is documented through literature [145]. In the 1995 research, the results showed that students have a particular preference for American TV series (e.g., *Beverley Hills, 90210* from 1900 to 2000 from FOX) [142], while in the 1997 research first for the Greek-Cypriot TV programs, subsequently for the Greek TV programs and finally for the American TV programs [143]. Also, in the 1995 research, the results showed that students watched TV for more than over 2 h a day (with a percentage of 63%) [142], while in the 1997 research, the students watched TV average 2 to 4 h a day (with a percentage of 85.9%) [143]. On the other hand, in the 2009 research in Greece, the results showed that students watched TV for at least 1 to 2 h a day (at a percentage of about 47%) [144]. Based on the specific conclusions, we can say that (a) the then Greek-Cypriot students were more familiar with TV (as audiovisual media communications) from the then Greek students as well as (b) the then Greek-Cypriot students consumed more audiovisual content through it from the then Greek students; who would probably still continue to behave the same way when they grow up, something that have been shown in the results this research (Table 2, Table 4, and Figure 4) and is confirmed through the findings and results of relevant research that took place in Greece (Thessaloniki) and Cyprus (Nicosia/Lefkosia) in 2016 with the sample consisting of young adults aged 18–25 years old as *digital natives* (now 22–29 years old/GenZ and Millennials) [131,133].

Also, from the analysis of the variation on two factors (2X2), Figure 4 shows statistically significant interaction between the gender of each country, while the analysis of the variation in three factors (2X3) in Figure 5 and Figure 6 show statistically significant effect in the age, something where they would be good to investigate further in later stage or to be investigated through other studies or/and based on the genealogical cohorts. Moreover, the results in Table 5 show that the answers from females are more positive in relation to the answers from males, which is a common phenomenon, especially in various research conducted in Cyprus and Greece in relation to the use of technology, new technologies, and ICTs in general in the last decade [1,2,28,144,146]. Finally, the results from the main research have shown that the venue, time, and period of a seminar (Table 3) are crucial and decisive factors for the implementation of a lesson, and this should be taken into account by adult educators and more studies could investigate. 

On the other hand, the results from this research, unfortunately, cannot allow their generalization to the population and certain limitations are imposed [80,81] in both researches, due to (a) the methodology, (b) the small number of the participants of the study (which was restricted to 40 and 76, respectively), and (c) the sampling method followed (Internet sampling was applied [47] in this case, as well as the relevant rules [77]); which was not the aim and purpose of this research anyway (but to provide data that will contribute to the effective teaching utilizing audiovisual media communications in education, and especially in adult education). The results are also not presented, for example, in double-entry tables in relation to specific demographics, such as age groups of the sample because they were not subject in both researches (but the adults only—without gender—as adult learners), something which would be good to investigate further at a later stage or to be investigated through other studies.

In conclusion, this research is part of a larger, ongoing research that explores the multidisciplinary field that incorporates MACE, ICTs in adult education (in Greece and Cyprus) which began in 2016, while part of its primary data was used as secondary data with other primary data of other researches for a secondary analysis [12,13]. The research protocol (including or/and the lesson plan) presented in this research can be applied to subsequent research or/and applied in a second step, with a larger group of participants that would deliver overall or/and generalized results against the population or/and base on the generational cohorts. Also, this research is considered as a case study with a mixed methodology [78,79] as a new methodological approach [44,45,46,47] (which is part of the new research methods [44,47]), and we hope that will become an important guide for those who apply this method or methods in their future researches, while its bibliography will become a source of further study. Finally, what we should bear in mind based on the results, is the correct use but also the appropriate choice of audiovisual content that we will use from and through audiovisual media technologies, because through it we can create non-verbal message, images, feelings, affects, emotions, and nostalgia [12,13,20,21,22,23,54,99,100,101,102,103,104]

## 6. Conclusions

We live in a world in which the development of some things is affected by interest, trust, and from non-verbal messages. Many sciences use this triple combination to find ways which they apply in order for improvement or change to take place [1,2,3], for example, those of media and education, and more specifically adult education [4,5,6,7,8,9,10]. The modern and visual way of life we are experiencing imposes an attitude of acceptance of innovations on us, as a result of living in a world which is constantly developing [1,2,3,27,33,34]. 

Effective integration of audiovisual media communications in adult education requires talented, dedicated, and committed adult educators with imagination, charisma, uniqueness, nervousness, patience, and perseverance who facilitate broadening the curriculum [4,5,6,7,15,16]. More specifically, if they implement a lesson plan from and through audiovisual media communications [4,5,6,7,15,16,17,18,19] should take into account the audiovisual content they will use as well as the importance of communication and behavior [12,13,22,23,92]. Also, they should keep in mind that the right selection of teaching methods utilizing audiovisual media communications should be adjusted based on the adult learners’ profile [15,16,24,25,26] and the genealogical characteristics of each generation [39,129,130].

The use of audiovisual media communications in the educational process on all educational levels and disciplines can generate motivation, stimulation of perceptual skills [15], and development of skills identified through 4C [14] that will lead to enhanced learning outcomes [15,27,28,124]. In addition, with the use of audiovisual media communications as educational techniques and tools, we can reduce the symptoms of fatigue and tiredness, improving concentration and helping learners in their psychological health through non-verbal communication [12,13,21,22,23]. Any type of audiovisual media communications must be customized and take into account the characteristics, culture, needs of the learners [4,5,15,24,25,71,72,135], and genealogical characteristics as *digital native* or *digital immigrant* [67,68], so that learning will never end and will be evidently lifelong.

In conclusion, the study and application of non-verbal communication is also crucial, especially if done with the use of audiovisual media communications. The function, utility, and knowledge of non-verbal communication in education, and especially in adult education, is essential, both in the real and in the virtual (digital) world to communicate better [23], and without conflict [22], from and through the audiovisual media communications [12,13].

## Figures and Tables

**Table 1:** Grouped total responses from the pilot survey.

Degree of Satisfaction	Very Poor	Poor	Fair	Good	Excellent	Mean	SD
Expectations	1–3%		5–13%	8–20%	26–65%	4.45	0.904
Organization		1–3%	5–13%	21–53%	13–33%	4.15	0.736
Interesting Suggestions		1–3%	4–10%	21–53%	14–35%	4.20	0.723
Development Issues			5–13%	19–48%	16–40%	4.28	0.679
Knowledge			5–13%	16–40%	19–48%	4.35	0.700
Educational Tools	1–3%	1–3%	3–8%	7–18%	28–70%	4.50	0.934

**Table 2:** Grouped total responses from the pilot survey by country of origin.

	Degree of Satisfaction	Very Poor	Poor	Fair	Good	Excellent	Mean	SD
CYPRUS	Expectations			1–7%	2–14%	11–79%	4.71	0.611
GREECE		1–4%		4–15%	6–23%	15–58%	4.31	1.011
CYPRUS	Organization			3–21%	5–36%	6–43%	4.21	0.802
GREECE			1–4%	2–8%	16–62%	7–27%	4.12	0.711
CYPRUS	Interesting Suggestions			1–7%	7–50%	6–43%	4.36	0.633
GREECE			1–4%	3–12%	14–54%	8–31%	4.12	0.766
CYPRUS	Development Issues			2–14%	5–36%	7–50%	4.36	0.745
GREECE				3–12%	14–54%	9–35%	4.23	0.652
CYPRUS	Knowledge			2–14%	5–36%	7–50%	4.36	0.745
GREECE				3–12%	11–42%	12–46%	4.35	0.689
CYPRUS	Educational Tools			1–7%	2–14%	11–79%	4.71	0.611
GREECE		1–4%	1–4%	2–8%	5–19%	17–65%	4.38	1.061

**Table 3:** Grouped total responses from the main research.

Degree of Satisfaction	Very Poor	Poor	Fair	Good	Excellent	Mean	SD
Expectations		2–3%	10–13%	50–66%	14–18%	4.00	0.653
Organization		2–3%	11–14%	40–53%	23–30%	4.11	0.741
Interesting Suggestions		2–3%	6–8%	40–53%	28–37%	4.24	0.709
Discussion Time	2–3%		16–21%	19–25%	39–51%	4.22	0.961
Questions / Answers	4–5%	2–3%	3–4%	13–17%	54–71%	4.46	1.064
Development Issues			10–13%	30–39%	36–47%	4.34	0.703
Knowledge		5–7%	16–21%	31–41%	24–32%	3.97	0.894
Time		3–4%	15–20%	43–57%	15–20%	3.92	0.744
Venue		3–4%	31–41%	31–41%	11–14%	3.66	0.776
Period		2–3%	13–17%	40–53%	21–28%	4.05	0.746
Educational Tools			12–16%	21–28%	43–57%	4.41	0.751

**Table 4:** Grouped total responses from the main research by country of origin.

	Degree of Satisfaction	Very Poor	Poor	Fair	Good	Excellent	Mean	SD
GREECE	Expectations		2–5%	5–13%	24–63%	7–18%	3.95	0.733
CYPRUS				5–13%	26–68%	7–18%	4.05	0.567
GREECE	Organization		2–5%	6–16%	24–63%	6–16%	3.89	0.727
CYPRUS				5–13%	16–42%	17–45%	4.32	0.702
GREECE	Interesting Suggestions		2–5%	2–5%	25–66%	9–24%	4.08	0.712
CYPRUS				4–11%	15–39%	19–50%	4.39	0.679
GREECE	Discussion Time	2–5%		10–26%	12–32%	14–37%	3.95	1.064
CYPRUS				6–16%	7–18%	25–66%	4.50	0.762
GREECE	Questions / Answers	4–11%	2–5%	3–8%	6–16%	23–61%	4.11	1.371
CYPRUS					7–18%	31–82%	4.82	0.393
GREECE	Development Issues			4–11%	16–42%	18–47%	4.37	0.675
CYPRUS				6–16%	14–37%	18–47%	4.32	0.739
GREECE	Knowledge		4–11%	10–26%	15–39%	9–24%	3.76	0.801
CYPRUS			1–3%	6–16%	16–42%	15–39%	4.18	0.943
GREECE	Time		2–5%	9–24%	22–58%	5–13%	3.79	0.741
CYPRUS			1–3%	6–16%	21–55%	10–26%	4.05	0.733
GREECE	Venue		2–5%	15–39%	14–37%	7–18%	3.68	0.714
CYPRUS			1–3%	16–42%	17–45%	4–11%	3.63	0.842
GREECE	Period		2–5%	8–21%	19–50%	9–24%	3.92	0.818
CYPRUS				5–13%	21–55%	12–32%	4.18	0.652
GREECE	Educational Tools			7–18%	9–24%	22–58%	4.39	0.790
CYPRUS				5–13%	12–32%	21–55%	4.42	0.722

**Table 5:** Grouped total responses from the main research by gender.

	Degree of Satisfaction	Very Poor	Poor	Fair	Good	Excellent	Mean	SD
MALES	Expectations		2–5%	7–19%	25–68%	3–8%	3.78	0.672
FEMALES				3–8%	25–64%	11–28%	4.21	0.570
MALES	Organization		2–5%	8–22%	19–51%	8–22%	3.89	0.809
FEMALES				3–8%	21–54%	15–38%	4.31	0.614
MALES	Interesting Suggestions		2–5%	4–11%	22–59%	9–24%	4.03	0.763
FEMALES				2–5%	18–46%	19–49%	4.44	0.598
MALES	Discussion Time	2–5%		9–24%	10–27%	16–43%	4.03	1.093
FEMALES				7–18%	9–23%	23–59%	4.41	0.785
MALES	Questions / Answers	3–8%	2–5%	1–3%	7–19%	24–65%	4.27	1.262
FEMALES		1–3%		2–5%	6–15%	30–77%	4.64	0.811
MALES	Development Issues			7–19%	15–41%	15–41%	4.22	0.750
FEMALES				3–8%	15–38%	21–54%	4.46	0.643
MALES	Knowledge		2–5%	11–30%	15–41%	9–24%	3.84	0.866
FEMALES			3–8%	5–13%	16–41%	15–38%	4.10	0.912
MALES	Time		3–8%	7–19%	22–59%	5–14%	3.78	0.787
FEMALES				8–21%	21–54%	10–26%	4.05	0.686
MALES	Venue		2–5%	15–41%	12–32%	8–22%	3.70	0.878
FEMALES			1–3%	16–41%	19–49%	3–8%	3.82	0.873
MALES	Period		2–5%	7–19%	18–43%	12–32%	4.03	0.866
FEMALES				6–15%	24–62%	9–23%	4.08	0.623
MALES	Educational Tools			9–24%	9–24%	19–51%	4.27	0.838
FEMALES				3–8%	12–31%	24–62%	4.54	0.643

**Table 6:** The data from the “FSS form”.

	FSS 1				FSS 2				Test-Retest	
Questions/Items ^1^	Score	%	Mean	SD	Score	%	Mean	SD	α	ICC ^2^
Q1	466	87.59	6.13	0.737	440	82.71	5.79	0.914	0.752	0.716
Q2	378	71.95	4.97	1.932	373	70.11	4.91	1.834	0.804	0.709
Q3	358	67,29	4.71	1.513	322	60.53	4.24	1.582	0.821	0.801
Q4	424	79.69	5.58	1.181	384	72.18	5.05	1.264	0.679	0.643
Q5	389	73,12	5.12	1.460	338	63.53	4.45	1.692	0.544	0.513
Q6	449	84,39	5.91	0.969	394	74.06	5.18	1.283	0.647	0.793
Q7	417	78,38	5.49	1.465	376	70.68	4.95	1.540	0.689	0.663
Q8	346	65.03	4.55	1.754	292	54.89	3.84	1.767	0.901	0.864
Q9	369	69.36	4.86	1.572	296	55.64	3.89	1.638	0.777	0.702
Average Total	3596	75.10	5.26		3215	67.15	4.69		0.876	

^1^Appendix D; ^2^ Intraclass correlation coefficient.

## Appendix A

WELCOME VIDEO|MY STORY video URL: https://youtu.be/acBr_Af8eVE.

## Appendix B

Non-Verbal Communication video URL: https://youtu.be/UeZN214AQts.

## Appendix C

GNTM 2|Italian widows and emotions video URL: https://youtu.be/EHIymDkM-EU.

## Appendix D—Questionnaire Items from the ‘’FSS form’’

Q1: My motivation is lower when I am fatigued

Q2: Exercise brings on my fatigue

Q3: I am easily fatigued

Q4: Fatigue interferes with my physical functioning

Q5: Fatigue causes frequent problems for me

Q6: My fatigue prevents sustained physical functioning

Q7: Fatigue interferes with carrying out certain duties and responsibilities

Q8: Fatigue is among my three most disabling symptoms

Q9: Fatigue interferes with my work, family or social life
